# Supplementary material for: Beyond safety drivers: Applying air traffic control principles to support the deployment of driverless vehicles
Source: PLoS One. 2020 May 21;15(5):e0232837. doi: 10.1371/journal.pone.0232837 (PMC7241796; doi:10.1371/journal.pone.0232837)
Supplement: Attachment — Submitted filename: PLOScomments.pdf [file pone.0232837.s001.pdf]

Response to Reviews for PONE-D-20-04402

Robert C. Hampshire, Shan Bao, Walter S. Lasecki, Andrew Daw, and Jamol Pender

First, we would like to extend our gratitude to the two reviewers and to the full editorial team for considering our work. We are delighted to see your interest in the paper, and we are grateful for the opportunity to incorporate your feedback into a new revision. Below, we respond to your comments and discuss how your insights have shaped the changes to this paper. Thank you again for your time and effort reviewing our work; we are excited to share this new version with you.

Response to Editor's Comments

1. "*Please ensure that your manuscript meets PLOS ONE's style requirements, including those for file naming.*"
   → Our manuscript is formatted in the provided PLOS ONE LaTeX template and adheres to the stated style requirements, including that our two figures follow the stated PLOS ONE convention for file naming.

2. "*We note that Figure 1 in your submission contain copyrighted images. All PLOS content is published under the Creative Commons Attribution License (CC BY 4.0), which means that the manuscript, images, and Supporting Information files will be freely available online, and any third party is permitted to access, download, copy, distribute, and use these materials in any way, even commercially, with proper attribution. For more information, see our copyright guidelines: http://journals.plos.org/plosone/s/licenses-and-copyright.*"
   → This figure is a demonstration image provided by the company Designated Driver. We have received explicit permission from the company for this image to be included in our paper. The permission form documenting this is attached to this revision.

Response to Reviewer's Comments

Reviewer #1: "*Very interesting paper and very good job. A nationwide remote monitoring system for driverless vehicles are proposed, which could increase safety dramatically, speed these vehicles' deployment, and provide employment. The remote operators do not directly drive the vehicles, but provide input on high level tasks such as path-planning, object detection and classification. No more comments.*"
→ Thank you, we appreciate your kind feedback and interest in our work!

Reviewer #2: "*This is an interesting paper, it is well written and easy to follow. I have the following comments:*"

(1) "*on Page 8, the authors assumed arrival rate is constant. This assumption, in my opinion, is kind of over simple. If we look at the distribution of crashes (assuming that number of crashes and remote drivers needed are proportional), it is not evenly distributed*

by hour, day (week day vs weekend), as well as other conditions (roadway type, weather, etc.). Taking the fallen tree as an example, significant more remote drivers may be needed under adverse weathers. I encourage the authors discuss it in the paper."
→ Thank you for raising this point. The study we have conducted here is a peak hour analysis that addresses the highest demand seen daily, thus treating the arrival rate as constant. Of course, it is very well-known that traffic intensities vary across time, so the issue is worth addressing. We have now added an additional subsection to Materials and Methods section that reviews how to extend these staffing calculations to time-varying settings. Some of our authors are researchers in this area, so we are able to make use of their expertise. Moreover, this also demonstrates that our peak hour analysis is justified, as the calculations are quite similar. In fact, these calculations reduce to the previous expressions when one considers the maximum rate across time. It is worth noting that this approach is akin to how we have calculated the time-varying staffing levels in Fig 2. Thank you for this suggestion, we believe this discussion has strengthened the paper.

(2) "*Lines 103 to 107 on Page 4, can you further clarify how you come up with the number of 6.25 m disengagements?*"
→ We have now added clarification on how this figure is reached, which is through aggregating the nationwide miles driven by each hour of the day and then converting from miles to disengagements through the product of the peak hour miles and the disengagement rate, which is 11,000 miles per disengagement.

(3) "*Minor: first line on Page 10. "Inter-arrival and service distributions" should be "Inter-arrival and service time distributions?" missing "time"?*"
→ Thank you for catching this, we have now clarified that this distribution refers to the service time specifically.

(4) "*One question not related to the work in this paper, but I am curious. How about if the remote driver makes mistakes and "causes" a crash? Should he/she take responsibility?*"
→ This is a great question, and we are very eager to see how it is eventually settled in practice. There are not many precedents we can draw upon, but perhaps air traffic control is again our best analogy. In this case, it seems that both the individual controllers and their employers can be held liable for mistakes. In such settings, it seems that the existence or absence of negligence is established on whether a well-trained expert would have been able to avoid the end result or if indeed it was an egregious error. While there are many differences between these settings, we would expect something similar to hold for autonomous vehicles. Employers may then offer to somewhat shield their remote operator employees from individual liability as a way of recruiting the best talent to the position. Of course, this response is again mostly speculative, as there is not yet a legal precedent for it. Nevertheless, it is fascinating to consider!
